# Use of Mobile Phone Text Message Reminders in Health Care Services: A Narrative Literature Review

**DOI:** 10.2196/jmir.3442

**Published:** 2014-10-17

**Authors:** Kati Anneli Kannisto, Marita Hannele Koivunen, Maritta Anneli Välimäki

**Affiliations:** ^1^Department of Nursing ScienceUniversity of TurkuTurkuFinland; ^2^Satakunta Hospital DistrictPoriFinland; ^3^Turku University HospitalTurkuFinland

**Keywords:** text messaging, short message service, cellular phone, reminder system, review

## Abstract

**Background:**

Mobile text messages are a widely recognized communication method in societies, as the global penetration of the technology approaches 100% worldwide. Systematic knowledge is still lacking on how the mobile telephone text messaging (short message service, SMS) has been used in health care services.

**Objective:**

This study aims to review the literature on the use of mobile phone text message reminders in health care.

**Methods:**

We conducted a systematic literature review of studies on mobile telephone text message reminders. The data sources used were PubMed (MEDLINE), CINAHL, Proquest Databases/ PsycINFO, EMBASE, Cochrane Library, Scopus, and hand searching since 2003. Studies reporting the use of SMS intended to remind patients in health services were included. Given the heterogeneity in the studies, descriptive characteristics, purpose of the study, response rates, description of the intervention, dose and timing, instruments, outcome measures, and outcome data from the studies were synthesized using a narrative approach.

**Results:**

From 911 initial citations, 60 studies were included in the review. The studies reported a variety of use for SMS. Mobile telephone text message reminders were used as the only intervention in 73% (44/60) of the studies, and in 27% (16/60) of the remaining studies, SMS was connected to another comprehensive health intervention system. SMS reminders were sent to different patient groups: patients with HIV/AIDS (15%, 9/60) and diabetes (13%, 8/60) being the most common groups. The response rates of the studies varied from 22-100%. Typically, the text message reminders were sent daily. The time before the specific intervention to be rendered varied from 10 minutes (eg, medication taken) to 2 weeks (eg, scheduled appointment). A wide range of different evaluation methods and outcomes were used to assess the impact of SMS varying from existing databases (eg, attendance rate based on medical records), questionnaires, and physiological measures. About three quarters of the studies (77%, 46/60) reported improved outcomes: adherence to medication or to treatment reportedly improved in 40% (24/60) of the studies, appointment attendance in 18% (11/60) of the studies, and non-attendance rates decreased in 18% (11/60) of the studies. Other positive impacts were decreased amount of missed medication doses, more positive attitudes towards medication, and reductions in treatment interruptions.

**Conclusions:**

We can conclude that although SMS reminders are used with different patient groups in health care, SMS is less systematically studied with randomized controlled trial study design. Although the amount of evidence for SMS application recommendations is still limited, having 77% (46/60) of the studies showing improved outcomes may indicate its use in health care settings. However, more well-conducted SMS studies are still needed.

## Introduction

With more than 6.8 billion mobile phone users and mobile phone technology penetration near 100% worldwide, mobile technology and text messages have changed communication between people [1] and increased the use of this technology in health care services [2]. Mobile phones are used in low-income countries [3,4] and in most social groups [2] including patients with psychiatric problems [5]. Due to its low costs, quick delivery [2], safety issues [6], and reduced intrusiveness compared to phone calls [5], mobile technology has been favored in various contexts and is recommended in a variety of strategies [7,8] and guidelines [9,10]. However, implementing new interventions requires continuous education and training among staff members [11].

The use of text messaging (short message service, SMS) applications for behavioral change is at an early stage of research [3]. Systematic reviews have already been conducted in this area, although discrepancies between the results of the previous reviews can be found. Previous reviews have shown that SMS reminders had a positive impact on patient appointment attendance [12], adherence to chronic medication [13] and to antiretroviral therapy [14], patient self-management [15] or health outcomes, and care processes [16]. On the contrary, Gurol-Urganci et al [17] found very limited evidence that communicating results of medical investigations by SMS would be useful. Kauppi et al [18] as well did not find clear evidence that information and communication technology (ICT)-based prompting (like SMS) would improve medication adherence with people with serious mental illness. However, little is known about which specific patient groups SMS reminders have been used for in health care. To form a more coherent picture of how SMS reminders have been used in clinical practice and to provide a more thorough understanding of the knowledge accumulated in the area, it is important to figure out the context, situations, and audience for past text message reminder use and the possible benefits to patients. Therefore, this review aims to synthesize studies investigating the use of mobile phone text message reminders in health care. The review was guided by the following questions: (1) What purposes have text message reminders been used for in health care?, (2) How have the impacts of text message reminders been assessed?, and (3) What are the impacts of using text messages as reminders in health care?

## Methods

### Design

A systematic review design with narrative methods was used. More precisely, a review methodology [19] was conducted to form a conception of the use of mobile phone text messages as reminders in health care.

### Search Strategy

We conducted a comprehensive literature search on February 21, 2013. The following electronic databases were searched with the help of an information specialist at the Medical Library: PubMed (MEDLINE), CINAHL, Proquest Databases/ PsycINFO, Embase, Scopus, and the Cochrane Library. The search terms (or equivalent index terms and free-text words) for each of the databases were used to ensure a broad coverage of published studies in our review. Detailed search terms are presented in Table 1.

References were also collected by screening the reference lists of the 906 articles, and 2 more papers were found. In addition, a hand search in all JMIR journals was conducted (in August 2014) leading to 3 additional papers [20-22]. Thus, we identified a total of 911 published articles relevant to our topic.

**Table 1:** Databases and search terms used, and references found (N=906).

Database	Search terms	References, n
PubMed (MEDLINE)	("Cellular Phone"[Mesh] OR "cell phone"[tiab] OR "cell phones"[tiab] OR "cellular phone"[tiab] OR "mobile phone"[tiab] OR "mobile phones"[tiab] OR "short message service"[tiab] OR "short messaging service"[tiab] OR "text messaging"[tiab] OR "text messages"[tiab] OR "text message"[tiab] OR (sms[tiab] AND (message[tiab] OR messages[tiab] OR messaging[tiab]))) AND ("Reminder Systems"[Mesh] OR remind*[tiab] OR prompt*[tiab])	315
CINAHL	(MH "Wireless Communications" OR MH "Telephone" OR MH "Instant Messaging" OR TI ("short message service" OR "short messaging service" OR "text messaging" OR "text messages" OR "text message" OR (sms AND (message OR messages OR messaging))) OR AB ("short message service" OR "short messaging service" OR "text messaging" OR "text messages" OR "text message" OR (sms AND (message OR messages OR messaging)))) AND (MH "Reminder Systems" OR TI remind* OR TI prompt* OR AB remind* OR AB prompt*)	194
Proquest Databases/ PsycINFO	all(reminder*) AND all("cellular phone*" OR sms OR "short text message*" OR "text messag*" OR "cell phone*" OR "mobile phone*")	75
Embase	((sms OR 'short text message' OR 'short text messages' OR 'text messages' OR 'text message' OR 'text messaging'/exp OR 'text messaging' OR 'mobile phone'/exp OR 'mobile phone' OR 'mobile phones' OR 'cell phone'/exp OR 'cell phone' OR 'cell phones' OR 'cellular phone'/exp OR 'cellular phone' OR 'cellular phones') AND ('reminder system'/exp OR 'reminder system')) OR 'sms reminder' OR 'sms reminders'	179
Scopus	(((sms OR "short text message*" OR "text messag*" OR "cell phone*" OR "mobile phone*" OR "cellular phone*") AND ("reminder system*")) OR "sms reminder*") AND (health)	143

### Eligibility Criteria

The review was limited to texts published in English, with an abstract available, between 2003 and 2013. The limitation in publication years was chosen due to a marked increase in information technology during the last decade [23]. The review was also limited to studies of text message reminders in the health care domain, sent from health care services to patients’ mobile phones. Patients of all ages and with any diagnoses were included. Further, we included only peer-reviewed, published papers using a variety of design and research methods.

Studies were excluded if SMSs were received by a parent, relative or friend, health care student, or staff member; if a reminder was sent by email or letter; or if SMS was used for non-clinical purposes (eg, for the recruitment of study participants, to survey patients’ willingness to receive text messages). Further, papers describing the design process of the SMS system, theoretical papers, statistical reviews, books or book chapters, letters, dissertations, editorials, and study protocols were excluded.

### Study Selection

**Figure 1:**
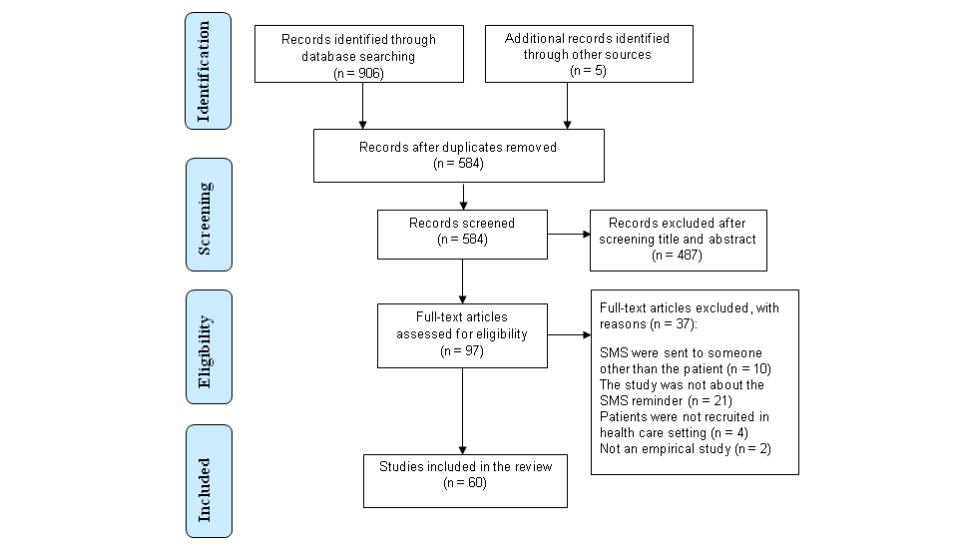
PRISMA flow diagram outlining the review process.

The study selection consisted of four steps. First, 2 authors (KK, MK) independently screened all titles and abstracts (n=911) of relevance for this systematic review [24]. Second, the abstracts of all relevant articles were screened for eligibility by the same 2 authors. Third, the full papers of the included publications were obtained and screened (KK) for inclusion and exclusion criteria. In case of any discrepancy between the decisions made, the papers were discussed until consensus was reached with the support of MV. Fourth, the reference lists of all papers included and systematic reviews identified in the original search were checked to find additional publications that met our inclusion criteria. After study selection, we had 60 studies to be extracted. Figure 1 outlines the search process of the literature [25].

### Data Extraction

We created a specific data extraction grid to collect information systematically related to our aims in the synthesis study by one author (KK). The data extraction was based on the CONSORT-EHEALTH checklist [26] where possible.

### Descriptive Characteristics of the Study

The following information of data items was collected [27]: (1) name(s) of the author(s), (2) year of publication (papers published since 2003 were included due to a marked increase in information technology during the last decade [23]), (3) country where the study was conducted, (4) purposes of the studies related to the use of mobile phone text message reminders, (5) setting of the studies was coded with a specific term used in the study (eg, outpatient or inpatient clinics, general or private hospitals), and the patient group who received text message reminders was extracted, (6) type (quantitative or qualitative) of the study, (7) study design, (8) number of patients enrolled and participating in the study, and response rate of the studies were extracted, (9) intervention based on mobile phone text message reminders, (10) goals of text messages, (11) dose of the intervention based on mobile phone text message reminders (eg, the number of text message reminders, how often text message reminders were sent), and the timing (eg, the time of day or week the text message was sent; the time of a reminder before a specific intervention) of the intervention were extracted, (12) outcome measures as a key concept used, (13) instruments used to measure the outcomes of the intervention based on mobile telephone text message reminders (eg, names of the instruments used), and (14) outcomes of the intervention were extracted and described as increased, decreased, or unchanged. Increase, decrease, or unchanged were then presented as arrows up (↑), down (↓) or horizontal (⇔) (respectively) (see [28]).

### Analyses

The data on each included study were entered into the specific data extraction grid. Each study was treated as a separate case. Descriptive characteristics of the studies were categorized manually according to our research questions. The methodological quality of the studies was appraised with the Mixed Method Appraisal Tool (MMAT) by Pluye et al [29]. The method was designed to appraise the methodological quality of the studies in complex systematic literature reviews that include qualitative, quantitative, and mixed methods studies. For appraising qualitative studies, we used Section 1 of the MMAT, which contains items related to data sources, data analysis, context, and researcher’s influence. Section 2 of the MMAT was used to appraise randomized controlled studies; it contains items related to randomization, allocation concealment, assessment of outcome data and completeness of follow-up (drop-out). Section 3 was used for non-randomized studies; it contains items related to participants’ recruitment, outcome measurements, comparability of groups, and completeness of outcome data. Section 4 was used for descriptive studies; it contains items related to relevant sampling strategy, representativeness of the sample, outcome measurements, and acceptability of the response rate. Each item was scored as “yes”, “no”, or “can’t tell” [29]. In 27% (16/60) of the studies, the quality score was 4/4, meaning that all four criteria were met. In 45% (27/60) of the studies, the quality score was 3/4. In 72% (43/60) of the included studies, the quality score was 3/4 or 4/4, indicating the methodological quality of the included studies.

## Results

### Study Selection

The literature search yielded 911 publications. Duplicates were removed leaving 584 papers for further abstract screening. Following that screening according to inclusion and exclusion criteria by the Centre for Reviews and Dissemination [24], we excluded 487 papers based on the title and the abstract. All together 97 potential articles were obtained for full-text review by 2 independent reviewers, of which 60 studies were included in the review for further data extraction.

### Characteristics of the Included Studies

**Figure 2:**
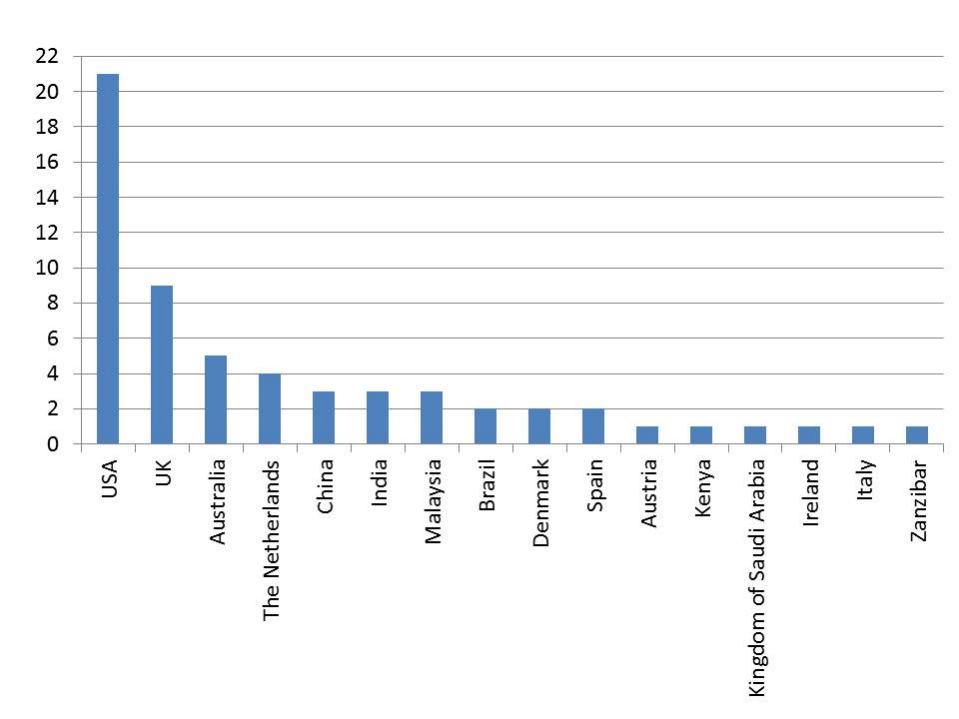
Countries of the publications included.

Author, year, country, setting, type of study, design, patient group, and sample were extracted to describe the characteristics of the studies. The authors of the studies are reported in each table dealing with the included studies (see Multimedia Appendices 1-3). The studies included in our analysis were published between 2004 and 2013. The number of published studies increased steadily until 2011, being highest in 2012. Of the included studies, 37% (22/60) studies were published in 2012. Except for one study [30], all publications involved outpatients. The studies were mostly conducted in the United States (35%, 21/60), followed by the United Kingdom and Australia (Figure 2).

Of the included studies, 95% (57/60) had a quantitative design, one (1) had a qualitative design, and two (2) used both quantitative and qualitative designs. Over one-third (35%, 21/60) of the studies were randomized controlled trials (RCT). Other studies were non-randomized feasibility studies, before-and-after studies, cross-sectional studies, retrospective and prospective studies, cohort studies with or without historical control, clinical trials, or qualitative descriptive studies.

The most common patient groups described were patients with human immunodeficiency virus/acquired immunodeficiency syndrome (HIV/AIDS; 15%, 9/60), diabetes (13%, 8/60), asthma (8%, 5/60), or schizophrenia (7%, 4/60). Other patient groups are described in more detail in Multimedia Appendix 2. The sample size of the studies varied from 4 participants to 9959. In half of the studies (53%, 32/60), the sample size was 100 or under, and in 23 studies (38%, 23/60), it was over 100. In 5 studies (8%, 5/60) the sample size was shown as the amount of appointments, not participants. The response rates of the studies varied from 22-100%. Descriptive characteristics of the included studies are presented in more detail in Multimedia Appendices 1 and 2.

### Purposes of Text Message Reminders Used in Health Care

The purpose of the study, description of the intervention, dose, and timing were extracted to describe the purposes of text message reminders used in health care. Mobile phone text message reminders were used to remind patients about their medication or treatment in 63% (38/60) of the studies, and both to increase the attendance to clinical appointments and to decrease the non-attendance to clinical appointments with patients from different patient groups in 37% (22/60) of the studies (Multimedia Appendix 2).

The mobile telephone text message reminders were used as the only intervention in 73% (44/60) of the studies (Multimedia Appendix 2). In 27% (16/60) of the studies, the approach was multifaceted, indicating that text message reminders were connected to another comprehensive health intervention system, such as educational text messages (7%, 4/60) [31-34], informational text messages about patients’ disease and healthy living (12%, 7/60) [20,21,32,33,35-37], supportive text messages [22,38] or to diary data collection [39]. In the studies by Rodrigues et al [40] and Sidney et al [41], reminders were sent as non-interactive neutral pictures delivered as an SMS.

The dose and timing of the text message reminders depended on the dosage of the medication or treatment and a patient’s scheduled appointment (Multimedia Appendix 2). In 55% (33/60) of the studies, the dose was reported as how often the text message reminder was sent, so that the most common dose was to send the text message reminders daily (35%, 21/60) [20-22,31,32,35,38,39,42-54]. In 38% (23/60) of the studies, the dose was reported as amount of text message reminders sent, and in 7% (4/60) of the studies, the dose was reported to depend on patients’ preferences.

The timing was reported as the time of the day (eg, morning, evening) or as a certain time (eg, 10 a.m., 8 p.m.) in 37% (22/60) of the included studies, and as how many days before the appointment the reminder was sent (eg, one day before the appointment) in 25% (15/60) of the studies . In 22% (13/60) of the studies, the timing was reported to be based on patients’ personal needs. However, in 17% (10/60) of the studies, the time of sending text messages was not reported. Further, the timing varied from 10 minutes before the medication was due to be taken [55] to 2 weeks before the scheduled appointment [56]. The dose and timing of the text message reminders are shown more specifically in Multimedia Appendix 2.

### Assessment Methods to Evaluate the Impact of Mobile Phone Text Message Reminders

A description of the instruments used as an assessment method was extracted (Multimedia Appendix 3). In 43% (26/60) of the studies, the impact was assessed using existing databases (eg, attendance rate/did not attend rate) [34,39,52,56-78].

In 12% (7/60) of the studies, the impact of the mobile phone text message reminders was assessed using questionnaires [20,32,41,49-51,53], and in 18% (11/60) of the studies, the impact was assessed using physiological measures [22,31,35,37,44,45,47,48,52,79,80]. Out of these 11 physiological assessments, patients’ self-assessment was used alone in one study, blood test alone in one study, self-reported weight in one study, and in eight studies patients’ self-assessment was connected with electronic monitoring, a questionnaire, pill counting, or a blood test. Other assessment methods were electronic monitoring alone (n=3) [42,46,81], pill count alone (n=1) [40], system usage calculation (n=1) [36], proportion of days covered calculation (n=1) [82], interview (n=1) [54], and observational measurement (n=2) [30,83].

In 10% (6/60) of the studies, the impact was assessed through patient satisfaction with the text message-based intervention [33,38,55,84-86]. In addition to these six studies, patient satisfaction was assessed in 15 studies. Patients’ satisfaction with the text message reminders was assessed in total in 20 studies, and patients’ reminder preferences in one study (Table 2). Patient satisfaction was assessed by questionnaires (n=14) [20-22,31-33,38,41,48,53,55,70,79,85], and by interviews (n=6) [36,39,44,73,80,84]. Patients’ preferences regarding reminders (n=1) were assessed by calculating the percentage of patients who selected the SMS reminders [86].

**Table 2:** Assessment of patient satisfaction.

Author (year)	Outcome measure		Instruments	Outcomes^a^
Anhøj & Møldrup (2004) [39]	Feasibility of using SMS for asthma diary data collection		Focus group interview	+
	Participants’ experiences with medication adherence reminders		Focus group interview	+
Agyapong et al (2013) [38]	Usefulness		Semistructured questionnaire	+
	Patient satisfaction with abstinence reminders		Semistructured questionnaire	+
	Patient satisfaction with medication reminders		Semistructured questionnaire	-
Arora et al (2012) [32]	Satisfaction with the TExT-MED program		Questionnaire	+
Branson et al (2011) [70]	Patient satisfaction with text message reminders		Questionnaire	+
Britto et al (2011) [85]	Usefulness		Questionnaire	+
	Acceptability		Questionnaire	+
da Costa et al (2012) [80]	Patient satisfaction		Interview	+
Dick et al (2011) [44]	Satisfaction with the text message-based program		Interview	+
Dowshen et al (2012) [48]	Feasibility		“Satisfaction survey”	+
	Acceptability		“Satisfaction survey”	+
Fischer et al (2012) [73]	Feasibility		Focus group interview	+
Furberg et al (2012) [21]	Patient satisfaction with text messages		Satisfaction survey via SMS	+
Greaney et al (2012) [86]	Automated reminder preferences		SMS calculation	28%
Hanauer et al (2009) [36]	Feasibility		Interview	+
Holtz & Whitten (2009) [84]	Feasibility		Interview	+
	Compliance with monitoring asthma		Log-in records	+
Kollman et al (2007) [79]	Feasibility and user acceptance		Questionnaire	+
Lewis et al (2013) [53]	Receptivity to adherence messaging		Message receptivity questions via two-way text messages	+
	Clinical outcomes		Blood test (total virus load and CD4 counts)	+
Lua et al (2012) [33]	Feasibility and acceptability		Feedback form	+
Mao et al (2008) [55]	Patient satisfaction		Standardized questionnaire	+
Nundy et al (2013) [20]	Feasibility and acceptability		Patient experience survey	+
Pena-Robichaux et al (2010) [31]	Usability and satisfaction of the TM system		Questionnaire	+
Shaw et al (2013) [22]	Feasibility and acceptability		Questionnaire	+
Sidney et al (2012) [41]	Usefulness		Structured questionnaire	+
	Reminder preference:		Structured questionnaire, data on the delivery	
		Voice reminder		87%
		SMS alone		11%

^a^+ patients’ positive feedback, - patients’ negative feedback.

### Impact of Using Text Messages as Reminders in Health Care

Outcome measures and outcomes were extracted to describe the impacts of using text messages as reminders in health care. Of the included studies, the outcome measures were adherence to medication or treatment (50%, 30/60), appointment attendance (22%, 13/60), appointment non-attendance (18%, 11/60), or patient satisfaction (10%, 6/60). The impacts of using SMS text messages as reminders in health care are described in Table 2 and Multimedia Appendix 3.

Out of 60 studies, the outcomes reportedly improved in 77% (46/60). First, adherence to medication or to treatment improved in 24 studies [20,21,31,32,35-37,39,40,43-46, 48-51,53,54,66,79-82]. Second, appointment attendance was reported to have improved in 11 studies [30,34,57,60,61,69,70,74,75,77,78]. Third, non-attendance rates reportedly decreased in 11 studies [56,59,62-65,67,68,71,72,76]. In addition, patients’ attitudes towards medication were reported to have improved [51], the number of missed medication doses reportedly decreased [44,81], and text messages were found to have reduced treatment interruptions [46].

Outcomes in patient satisfaction were positive in those studies (n=6) where no impact was assessed [33,38,55,84-86]. In addition to these studies, patient satisfaction was assessed in 15 studies together with the impact assessment. In patients’ opinions, text messages were easy to use [20,44,48], they reminded patients to take their medication [32], patients were willing to receive text messages [53,66], and they were satisfied with text messages [69].

Using text messages had advantages over other reminding systems. Text messages could be sent to patients simultaneously, they were always available [59], cost-effective [59,63], and sending text messages to patients required less staff [63]. Liew et al [65] found that text messages were as effective as telephone reminders but were low-priced [60,61]. However, in the study by Greaney et al [86], participants preferred automated voice response reminders (72%) instead of SMS reminders (28%). Patients’ opinions about the usefulness of the text messages received varied from 88% [31] to 66% [81].

However, daily text message reminders did not improve adherence to oral contraceptive pills [42], acne treatment [47], or lupus erythematosus treatment [52]. Pijnenborg et al [30,83] found that the overall effect of prompting disappeared after the text message reminders ceased, indicating the dependence on continuous use of the intervention. Bos et al [58] and Fischer et al [73] found that there were no differences in appointment attendance before and after sending text message reminders.

Despite all the benefits and beneficial characteristics of the mobile phone text message reminders, this literature review shows that there are limitations to using mobile phone text message reminders. First, patients had privacy concerns about losing their mobile phones and other people possibly gaining access to the messages [67,70]. Although most (93%, 56/60) of the studies reported that the messages did not include the patient’s name or other identification in the reminder message, four exceptions were found [47,60,61,72]. No adverse events were reported. Second, patients may have changed their mobile phone numbers without informing the health care staff [60,61,67], thus the staff could not be sure that all participants had received the text messages. Downing et al [77] found that the proportion of undelivered text messages was high. Koshy et al [63] demonstrated that patients may have not received the text message reminders due to incorrect data entry. Third, it is possible that patients adapted to the messages and stopped reading them [43].

## Discussion

### Principal Findings

The results of this narrative literature review showed that mobile phones and text messages are used worldwide, which supports the global penetration of mobile phone subscriptions [1] in different user groups in health care [2,12,14,15]. We have demonstrated that mobile phone text messages may have their uses in reminding patients about medication adherence [43,45,47,50] and in reducing non-attendance rates [59,61,63,76]. The possibility of using text message reminders as the only intervention or in conjunction with some other comprehensive health intervention systems further adds to the usability of text messages in health care services. Thus, SMS reminders deserve more attention as a potential innovation to improve health care operations [87].

On the other hand, some concerns were also identified. First, in 4 studies, the dose of text message reminders (eg, the number of text message reminders, how often text message reminders were sent), and in 12 studies, the timing of the text message reminders reported were based on patients’ personal needs. Second, despite the safety of the text messages in health care [6], the literature review demonstrated privacy concerns, such as loss of a mobile phone or other people reading the messages [67,70]. Therefore, more emphasis should be put on how to guarantee that health-related patient information in electronic systems is anonymous and neutral enough to be managed even in open electronic systems. Special considerations are also required in designing the content of the reminder messages, entering the patient data to the automatic systems or dialing correct mobile phone numbers to protect patients’ privacy and security issues [4,88].

The impacts of text message reminders focused on improving adherence to medication and improving appointment attendance. Although no meta-analysis was used due to the high heterogeneity of the data gathered, this review demonstrated that text message reminders were easy to use, useful for patients, they were willing to receive text messages, and satisfied with the text message reminders. This knowledge is essential because patients’ views influence the acceptance of the text message intervention and its integration into patients’ daily lives [13]. On the other hand, patients may adapt to the messages and the effectiveness of the messages diminishes. This is what happened in the study by Strandbygaard et al [43]; participants in the intervention group stopped reading their reminder messages after a few weeks.

### Limitations

We recognize that there are some limitations in our review. First, the literature search yielded studies that were diverse methodologically and clinically. As the studies included were heterogeneous in study design, patient group studied, sample size, description of the intervention, and outcome measures, we synthesized the data with a narrative method, rather than trying to do a meta-analysis. As such, our findings cannot be used to recommend any preferred strategy for the use of mobile phone text message reminders in health care. Second, the studies differed in their methodological quality, which may have had an impact on the results, and biased our findings and limited our interpretations. Third, we included studies only from peer-reviewed English-language journals, which may have restricted our findings and biased the data toward positive results. And fourth, we excluded studies if text messages were received by parents, relatives, or friends, which may exclude a wide variety of studies (eg, immunization reminders; see [89,90]) in different fields and further affected the conclusions from the review.

### Future Research

Further evaluation of mobile phone text message reminder interventions is needed to form a more coherent picture of their use and effectiveness in health care services. This should be done with rigorous RCT studies of their effectiveness and cost-effectiveness. The research should also focus more on service users’ and their caregivers’ needs and preferences regarding the text message reminders to be received and how to maintain interest in text message reminders to achieve the best possible impact. In addition, the assessment of users’ satisfaction toward intervention should also be ensured. In this task, qualitative evaluations could also be used to hear users’ voices. More research is also needed to ascertain the best ways to guarantee privacy and security in mobile phone text message reminder interventions.

### Conclusions

The findings of this literature review are encouraging. However, the amount of evidence for SMS application recommendations is still limited. In our review, having 77% (46/60) of the studies showing improved outcomes may still indicate its use in health care settings. Although no firm conclusions can be drawn so far, mobile phone text message reminders may be a potential method in health care systems. Given the widespread use of mobile phone text message reminders among different patient groups, it may have the potential to improve adherence to medication and attendance at clinical appointments globally.

## Multimedia Appendix 1

Descriptive characteristics of the included studies.

## Multimedia Appendix 2

Population, sample size, response rates, description of the intervention, goal of the text messages, dose, and timing of the SMS intervention.

## Multimedia Appendix 3

Assessment methods and outcomes.

## Abbreviations

ICT: information and communication technology
MMAT: Mixed Method Appraisal Tool
RCT: randomized controlled trial
SMS: short message service
