# Supplementary material for: Use of Mobile Phone Text Message Reminders in Health Care Services: A Narrative Literature Review
Source: J Med Internet Res. 2014 Oct 17;16(10):e222. doi: 10.2196/jmir.3442 (PMC4211035; doi:10.2196/jmir.3442)
Supplement: Supplementary file 1 [file jmir_v16i10e222_app1.pdf]

Author (year)	Country	Purpose of study	Setting<br>Patient group	Type of study	Design	Quality appraisal (MMAT)
**Anhøj & Møldrup (2004) [39]**	Denmark	To evaluate the feasibility of using SMS for asthma diary data collection and patient compliance with an SMS diary	Outpatient clinic<br><br>Patients with asthma	Quantitative and qualitative	Mixed methods	3/4
**Agyapong et al (2013) [38]**	Ireland	To examine the perception of patients with AUD and comorbid depression about the usefulness of supportive text messages	University psychiatric hospital<br><br>Patients with alcohol use disorder	Quantitative	Cross-sectional semi-structured survey	3/4
**Altuwaijri et al (2012) [72]**	Kingdom of Saudi Arabia	To evaluate the effect of sending SMS reminders to patients with outpatient clinic appointments on non-attendance rates	Outpatient clinic<br><br>Outpatients	Quantitative	Retrospective data-analysis study	3/4
**Arora et al. (2012) [32]**	USA	To assess satisfaction and preliminary effectiveness of the "TExT-MED program"	Emergency department<br><br>Patients with diabetes	Quantitative	Prospective proof-of-concept study	2/4
**Balato et al (2012) [37]**	Italy	To evaluate the use of SMS in improving treatment adherence and other patient outcomes with patients with psoriasis	Department of Dermatology<br><br>Patients with psoriasis	Quantitative	RCT	4/4
**Boker et al (2012) [47]**	USA	To determine if daily automated SMS would result in increased adherence to recommended use of topical acne medication	Dermatology clinics<br><br>Patients with acne	Quantitative	RCT	3/4
**Bos et al (2005) [58]**	The Netherlands	To retest the hypothesis that a reminder would reduce the failed attendance rate	Orthodontic clinic<br><br>Patients at orthodontist clinics	Quantitative	RCT	2/4

**Bourne et al (2011) [69]**	Australia	To evaluate the impact of an SMS system on HIV/sexually transmitted infection re-testing rates among men who have sex with men	Sexual health clinic<br><br>Patients with HIV/ sexually transmitted infection	Quantitative	Cohort study	2/4
**Brannan et al (2011) [56]**	UK	To determine the effectiveness of an SMS reminder in improving attendance in return general ophthalmology clinic patients	Ophthalmology clinic<br><br>Patients at ophthalmology clinic	Quantitative	Prospective study	3/4
**Branson et al (2011) [70]**	USA	To examine the feasibility, acceptability, and preliminary efficacy of text message appointment reminders to improve attendance in outpatient therapy	Outpatient child mental health clinic<br><br>Adolescents with mental health problems	Quantitative	Quasi-experimental	4/4
**Britto et al (2011) [85]**	USA	To assess the feasibility, acceptability and utility of a text messaging system on teenagers with asthma	Pediatric academic medical center<br><br>Patients with asthma	Quantitative	Nonrandomised feasibility trial	3/4
**Chen et al (2008) [61]**	China	To compare the efficacy of an SMS reminder and phone reminder to improve attendance rates	Health promotion center<br><br>Outpatients	Quantitative	RCT	3/4
**da Costa et al (2010) [67]**	Brazil	To evaluate the impact of appointment reminders sent as SMS on nonattendance rates	Outpatient clinic<br><br>Outpatients	Quantitative	Cohort study	3/4
**da Costa et al (2012) [80]**	Brazil	To assess whether a warning system based on mobile SMS messages increases the adherence of HIV-infected women to antiretroviral treatment	Center for infectious diseases in pregnancy<br><br>Patients with HIV/AIDS	Quantitative	RCT	3/4

**Dick et al (2011) [44]**	USA	To develop and assess the feasibility of a diabetes-focused text message-based reminder and data collection system	Outpatient clinic<br><br>Patients with diabetes	Quantitative	Pre-and-post design	3/4
**Downer et al (2005) [59]**	Australia	To evaluate the effect of appointment reminders sent as SMS text messages on attendance at outpatient clinics	Children's Hospital<br><br>Outpatients	Quantitative	Cohort study with historical control	2/4
**Downing et al (2013) [77]**	Australia	To assess the effectiveness of using SMS reminders with and without incentive payments to increase re-testing rates in clients diagnosed with Chlamydia	Sexual health clinic<br><br>Patients with chlamydia	Quantitative	RCT	4/4
**Dowshen et al (2012) [48]**	USA	To evaluate the feasibility, acceptability and preliminary efficacy of SMS reminders to improve adherence to ART for youth living with HIV/AIDS	Community based health center<br><br>Patients with HIV/AIDS	Quantitative	Prospective, pre-post design	3/4
**Fairhurst & Sheikh (2008) [62]**	UK	To evaluate the effectiveness of texting appointment reminders to patients who persistently fail to attend appointments	Inner city general practice<br><br>Outpatients	Quantitative	RCT	4/4
**Fischer et al (2012) [73]**	USA	To assess the feasibility of engaging adults with diabetes in self-management behaviors between clinic visits by using cell phone text messaging to provide blood sugar measurement prompts and appointment reminders	Community health center<br><br>Patients with diabetes	Quantitative	Quasi-experimental	3/4
**Foley & O'Neill (2009) [64]**	UK	To evaluate the operational and financial efficacy of sending short message service (SMS) mobile telephone text message to patients with outpatient clinic appointments	Pediatric dental outpatient clinic<br><br>Outpatients	Quantitative	Cohort study with historical control	2/4
**Foreman et al (2012) [82]**	USA	To evaluate medication adherence among patients opting to receive text message medication reminders	Outpatient clinic<br><br>Patients with chronic disease	Quantitative	Cohort study	3/4

**Franklin et al (2006) [35]**	UK	To assess a text-messaging support system in pediatric patients with Type 1 diabetes	Outpatient clinic<br><br>Patients with diabetes	Quantitative	RCT	4/4
**Furberg et al (2012) [21]**	USA	To develop, implement and test a tailored SMS-based intervention for HIV-positive patients	Primary care clinic<br><br>Patients with HIV	Quantitative	Proof-of-concept study	1/4
**Granholm et al (2012) [49]**	USA	To pilot test an interactive text-messaging intervention for medication adherence, socialization, and auditory hallucinations	Community-dwelling<br><br>Patients with schizophrenia	Quantitative	Quasi-experimental	1/4
**Greaney et al (2012) [86]**	USA	To examine the association between participants' characteristics and preferred reminder modality	Urban health center<br><br>Outpatients	Quantitative	RCT	2/4
**Guy et al (2013) [78]**	Australia	To evaluate the impact of an SMS reminder system on chlamydia re-screening rates among women and heterosexual men	Public sexual health clinic<br><br>Patients with chlamydia	Quantitative	Before-and-after study	3/4
**Hanauer et al (2009) [36]**	USA	To test the feasibility of implementing a fully automated, two-way text messaging system to encourage increased blood glucose monitoring	Diabetes center<br><br>Patients with diabetes	Quantitative	Quasi-experimental	3/4
**Hardy et al (2011) [45]**	USA	To compare the efficacy of a personalized cell phone reminder system in enhancing adherence to ART to a beeper with patients with HIV	Outpatien HIV clinic<br><br>Patients with HIV	Quantitative	RCT	3/4
**Holtz & Whitten (2009) [84]**	USA	To determine the feasibility of monitoring asthma via an SMS application	Outpatient clinic<br><br>Patients with asthma	Quantitative	Quasi-experimental	1/4

**Hou et al. (2010) [42]**	USA	To estimate daily text-message reminders impact on oral contraception pill adherence	Planned Parenthood clinic<br><br>Oral contraceptive pill users	Quantitative	RCT	4/4
**Kollman et al (2007) [79]**	Austria	To evaluate the feasibility and user acceptance of a mobile-based data service to support diabetes	Diabetes clinic<br><br>Patients with diabetes	Quantitative	Clinical pilot trial	4/4
**Koshy et al (2008) [63]**	UK	To assess the effectiveness of the use of SMS-based reminders for hospital outpatient appointments as a method of reducing the non-attendance rates	Hospital outpatient ophthalmology clinic<br><br>Patients attending outpatient ophthalmology clinics	Quantitative	Observational study	3/4
**Leong et al (2006) [60]**	Malaysia	To determine the effectiveness of a text messaging reminder in improving attendance in primary care	Primary care clinics<br><br>Primary care patients	Quantitative	RCT	4/4
**Lewis et al (2013) [53]**	USA	To determine if dynamically tailored medication messages delivered to people living with HIV via text messaging would be well received and enhance adherence and clinical outcomes	Urban health clinic<br><br>Patients with HIV	Quantitative	One-group prepost test preexperimental design	4/4
**Liew et al (2009) [65]**	Malaysia	To determine if text messaging would be effective in reducing non-attendance in patients on long-term follow-up	Urban, primary care clinics for patients requiring chronic disease care<br><br>Patients with chronic disease	Quantitative	RCT	4/4

**Lua et al (2012) [33]**	Malaysia	To develop and assess the feasibility and acceptability of an SMS-based epilepsy educational program	General hospitals neurology clinic<br><br>Patients with Epilepsy	Quantitative	Prospective randomized interventional study	4/4
**Ludlow et al (2009) [66]**	UK	To investigate whether the use of email and text messaging to remind patients to have blood tests might result in better compliance than using more conventional methods of communicating	University hospital<br><br>Patients with inflammatory bowel disease	Quantitative	Quasi-experimental	2/4
**Lund et al (2012) [34]**	Zanzibar	To examine the association between a mobile phone intervention and skilled delivery attendance in a resource-limited setting	Primary health care facilities<br><br>Pregnant women	Quantitative	RCT	3/4
**Lv et al (2012) [50]**	China	To know whether SMS can improve perceived control of asthma	Department of Respiratory Medicine<br><br>Patients with asthma	Quantitative	RCT	1/4
**Mao et al (2008) [55]**	China	To develop a mobile pharmacy service system (MPSS) to deliver individualized pharmaceutical care via the SMS, with the aim of improving medication compliance and safety	General hospital<br><br>Outpatients	Quantitative	Quasi-experimental	2/4
**Milne (2010) [68]**	UK	To test the effectiveness of patient-focused booking and last minute reminders using SMS messages to reduce non-attendance at consultant outpatient clinics	Consultant outpatient clinics<br><br>Patients needing specialist medical advice and treatment from consultants	Quantitative	Cohort study	2/4

**Montes et al (2012) [51]**	Spain	To assess the impact of SMS-based strategy on adherence to antipsychotic treatment	Outpatietnt psychiatric centre<br><br>Patients with schizophrenia	Quantitative	RCT	4/4
**Nundy et al (2013) [54]**	USA	To explore the potential mechanism by which a text-message based diabetes program affected self-management	Academic medical center<br><br>Patients with diabetes	Qualitative	Qualitative description	3/4
**Nundy et al (2013) [20]**	USA	To assess the feasibility and acceptability of SMS-base intervention and explore its effects on self-management	Academic medical center (Cardiology services)<br><br>Patients with acute decompensated heart failure	Quantitative	Pretest and posttest	3/4
**Pena-Robichaux et al (2010) [31]**	USA	To evaluate the use of text messages to provide treatment adherence reminders and patient education in adults and adolescents with atopic dermatitis	General hospital<br><br>Patients with atopic dermatitis	Quantitative	Pretest and posttest	3/4
**Perry (2011) [71]**	UK	To assess the use of Short Message Service (SMS) text reminders on appointment attendance rates at a dental access centre	Dental access centre<br><br>Patients at dental access centre	Quantitative	Before and after design	2/4
**Pijnenborg et al (2007) [30]**	The Netherlands	To evaluate the efficacy of short message service (SMS) text messages as a compensatory aid to improve independence in individuals diagnosed with schizophrenia and cognitive deficits	Department of psychotic disorders: inpatients<br><br>Patients with schizophrenia	Quantitative	Single case experimental design	2/4

**Pijnenborg et al (2010) [83]**	The Netherlands	To evaluate the efficacy of short message service (SMS) text messages to compensate for the effects of cognitive impairments in schizophrenia in daily life	Department of psychotic disorders: inpatients and outpatients<br><br>Patients with schizophrenia	Quantitative	Waiting list controlled trial	3/4
**Pop-Eleches et al (2011) [46]**	Kenya	To test the efficacy of SMS reminders on adherence to ART	HIV clinic<br><br>Patients with HIV	Quantitative	RCT	3/4
**Prasad & Anand (2012) [74]**	India	To evaluate the effect of SMS appointment reminders on attendance at outpatient clinics	Dental outpatient clinic<br><br>Patients at dental clinics	Quantitative	RCT	2/4
**Rodrigues et al (2012) [40]**	India	To assess the influence of mobile phone reminders on adherence to antiretroviral therapy	Infectious disease clinic<br><br>Patients with HIV	Quantitative	Quasi-experimental cohort study	3/4
**Shaw et al (2013) [22]**	USA	To develop and test feasibility and acceptability of an SMS-based intervention to promote sustaining recent weight loss	Diet and fitness center<br><br>Obese participants	Quantitative	Experimental study	2/4
**Sidney et al (2012) [41]**	India	To assess the perceived usefulness and acceptability of mobile phone reminders to support adherence to ART	Infectious disease clinic<br><br>Patients with HIV	Quantitative	Cross-sectional study	4/4
**Sims et al (2012) [75]**	UK	To examine the effect of SMS reminders on the attendance of appointments at mental health clinics	Community mental health clinics<br><br>Mental health outpatients	Quantitative	Cohort study	4/4
**Strandbygaard et al (2010) [43]**	Denmark	To examine the impact of receiving daily text message reminders on the adherence to asthma treatment	Outpatien clinic<br><br>Patients with asthma	Quantitative	RCT	3/4

**Taylor et al (2012) [76]**	Australia	To investigate whether SMS reminders reduce non-attendance in physical therapy outpatient clinics	Physical therapy outpatient clinic<br><br>People in a physical therapy outpatient clinic	Quantitative	RCT	4/4
**Ting et al (2012) [52]**	USA	To investigate the effects of cellular text messaging reminders on adherence to clinic visits and medication among adolescents and youth with systemic lupus erythematosus	Pediatric rheumatology clinic<br><br>Patients with childhood-onset systemic lupus erythematosus	Quantitative	Quasi-experimental	4/4
**Vervloet et al (2012) [81]**	The Netherlands	To investigate the effect of SMS reminders on adherence to oral antidiabetics	Pharmacies<br><br>Patients with diabetes	Quantitative	RCT	3/4
**Vilella et al (2004) [57]**	Spain	To evaluate whether a reminder of the next vaccination dose sent by the Short Message Service (SMS) increases compliance with hepatitis A+B and hepatitis A vaccination schedule	Vaccination centre<br><br>Travellers	Quantitative	Experimental, controlled study, historical control	3/4

SMS=Short Message Service
MMAT: for example 3/4=3 out of for 4 criterion met
