# Supplementary material for: Use of Mobile Phone Text Message Reminders in Health Care Services: A Narrative Literature Review
Source: J Med Internet Res. 2014 Oct 17;16(10):e222. doi: 10.2196/jmir.3442 (PMC4211035; doi:10.2196/jmir.3442)
Supplement: Supplementary file 2 [file jmir_v16i10e222_app2.pdf]

Author (year)	Population Sample size (response rate %)	Description of the intervention	Goal of the text message	Dose	Timing
**Anhøj & Møldrup (2004) [39]**	15<br>12<br>(80%)	Automated SMS messages: including a medication reminder, and 3 diary questions	Medication reminder	4 SMS messages daily	At a participant's self-selected time of the day
**Agyapong et al (2013) [38]**	26<br>24<br>(92%)	Supportive text messages, eg promoting adherence with medication and abstinence from alcohol	Supportive text message	Twice daily	At 10.00 and 19.00
**Altuwaijri et al (2012) [72]**	N/a<br>Total not described<br>N/a	Automated SMS messages	Appointment reminder	1 text message	5 days and a day prior to the appointment date
**Arora et al. (2012) [32]**	23<br>23<br>(100%)	Automated text messages from 5 categories: 1) educational and motivational, 2) medication reminders (3 times per week), 3) healthy living challenges, 4) trivia, and 5) phone links	Medication reminder	2 SMS messages per day or one SMS message 1-3 per week	At 9 a.m., 12 p.m., and 6 p.m.
**Balato et al (2012) [37]**	40<br>40<br>(100%)	Manually sent text messages	Treatment reminder	Reminders 3 times weekly: 1 text message per day	Not described
**Boker et al (2012) [47]**	40<br>33<br>(83%)	Automated and customized text messages reminding about their medication	Medication reminder	Twice daily	In the morning and evening
**Bos et al (2005) [58]**	343<br>301<br>(88%)	A reminder either by telephone, mail or SMS	Appointment reminder	1 text message	A day before the appointment
**Bourne et al (2011) [69]**	N/a<br>3551<br>N/a	A SMS reminder template added to the patient electronic database	Appointment reminder	1 text message	Not described

**Brannan et al (2011) [56]**	N/a<br>201<br>N/a	A customized text message reminder	Appointment reminder	1 text message	Two weeks before the scheduled appointment
**Branson et al (2011) [70]**	48<br>48<br>(100%)	Manually sent text message reminder	Appointment reminder	1 text message	The evening before each scheduled appointment
**Britto et al (2011) [85]**	23<br>19<br>(83%)	Web-based calendar-to-text system for appointment and medication reminders: patients were able to create their own messages	Appointment and medication reminders	Participants were able to determine how often the messages were sent	At a participant's self-selected time of the day
**Chen et al (2008) [61]**	1859<br>1848<br>(99%)	SMS or telephone call reminder	Appointment reminder	1 text message	72 h prior to the appointment
**da Costa et al (2010) [67]**	N/a<br>32709[a]<br>N/a	Automated SMS text message	Appointment reminder	1 text message	24h before the appointment
**da Costa et al (2012) [80]**	29<br>21<br>(72%)	Automated SMS message	Medication reminder	On Saturdays and Sundays and during the working week on alternate days	30 min before the required time of the last required medication dose in a day
**Dick et al (2011) [44]**	19<br>18<br>(95%)	Automated text messages	Treatment adherence	Daily (medication or blood sugar reminder), weekly (question about foot care)	Not described
**Downer et al (2005) [59]**	N/a<br>2864<br>N/a	Automated SMS reminder	Appointment reminder	1 text message	3 working days before the appointment date
**Downing et al (2013) [77]**	94<br>76<br>(81%)	Manually sent SMS reminders	Appointment reminder	1 text message	On Mondays
**Dowshen et al (2012) [48]**	25<br>21<br>(84%)	Automated, personalized SMS reminder messages	Medication reminder	Daily	At the time(s) specified by patient

**Fairhurst & Sheikh (2008) [62]**	N/a<br>172<br>N/a	Text message reminder of the appointment	Appointment reminder	1 text message	08:00-09:00 a.m. or 04:00-05:00 p.m.
**Fischer et al (2012) [73]**	N/a<br>47<br>N/a	Automated text message prompts about blood sugar readings and appointment reminder messages	Appointment reminder	3 times a week (blood sugar) and 3 times advance each appointment	At 7:15 a.m. on Mondays, Wednesdays and Fridays (blood sugar) and at 7, 3 and 1 day(s) prior to the scheduled appointment
**Foley & O'Neill (2009) [64]**	N/a<br>709<br>N/a	Automated SMS reminders	Appointment reminder	1 text message	24 h prior to their appointment
**Foreman et al (2012) [82]**	580<br>580<br>(100%)	Text message reminders about medication (6 categories)	Medication reminder	Varies according to the patients	Any hour of the day
**Franklin et al (2006) [35]**	92<br>90<br>(98%)	Automated delivery of a series of appropriately tailored messages, including a weekly reminder of the goal set in clinic	Treatment reminder	Daily (tips) or weekly (reminder)	Not described
**Furberg et al (2012) [21]**	52<br>46<br>(88%)	SMS reminders and supportive messages to enhance outcomes related to managing HIV	Appointment and medication reminders	Appointment: within 3 days prior to appointment<br>Medication: daily	Appointment: randomly selected<br>Medication: according to participants clinical dosing time
**Granholm et al (2012) [49]**	55<br>42<br>(76%)	Text messages focusing on 3 intervention domains: medication adherence, socialization and auditory hallucinations	Medication reminder	3 sets of 4 text messages (12 total) daily	In the morning, afternoon and evening
**Greaney et al (2012) [86]**	N/a<br>598<br>N/a	Automated electronic reminders (SMS or automated voice response messages) with participants selecting modality	Supportive text messages	1 text message	Every other week
**Guy et al (2013) [78]**	N/a<br>681<br>N/a	Automated text message reminders	Appointment reminder	1 text message	On the prespecified date set

**Hanauer et al (2009) [36]**	40<br>29<br>(73%)	Automated reminders either via cell phone text messaging or by e-mail	Treatment monitoring reminder	The number of reminders were determined by the participants	The frequency of reminders were determined by the participants
**Hardy et al (2011) [45]**	23<br>19<br>(83%)	Automated, personalized text messages	Medication reminder	Daily	Matching the ART dosing frequency
**Holtz & Whitten (2009) [84]**	N/a<br>4<br>N/a	Automated text message reminder, if the patient did not sent a peak flow reading	Treatment adherence	1 text message	At 11 a.m.
**Hou et al. (2010) [42]**	82<br>73<br>(89%)	Automated text message reminders	Medication reminder	Daily	At a designated time chosen by the participant
**Kollman et al (2007) [79]**	10<br>10<br>(100%)	Mobile phone-based diabetes management system, including text message reminders	Treatment adherence	SMS was sent if less than 3 blood glucose measurements had been received that day	In the evening
**Koshy et al (2008) [63]**	N/a<br>9959<br>N/a	Automated SMS reminder	Appointment reminder	1 text message	One day or four days before the appointment
**Leong et al (2006) [60]**	993<br>964<br>(97%)	Text message and mobile phone call reminders	Appointment reminder	1 reminder	24-48 hours prior to the scheduled appointment
**Lewis et al (2013) [53]**	52<br>46<br>(88%)	Automated, tailored text message	Medication reminder	1-3 times per day	Based on the participants' own ART medication regimen
**Liew et al (2009) [65]**	931<br>769<br>(83%)	Reminders via text messaging or telephone call	Appointment reminder	1 reminder	24-48 hours prior to the appointment

**Lua et al (2012) [33]**	51<br>51<br>(100%)	SMS-based educational system sending text messages to patients' mobile telephone including educational messages, drug-taking reminders and clinic reminders	Appointment and medication reminders	Drug-taking reminder once a month, appointment reminder a day before the appointment	At 10:00 a.m.
**Ludlow et al (2009) [66]**	126<br>126<br>(100%)	A reminder via email, text messaging or telephone call (control group)	Treatment monitoring reminder	The week before the blood test was due, the week the test was due and the following week, if no blood test results were available	On Monday morning
**Lund et al (2012) [34]**	2637<br>2550<br>(97%)	An automated SMS system providing (educational messages and appointment reminder) and mobile phone voucher component throughout the pregnancy and 6 weeks after delivery	Appointment reminder	At the beginning two messages a month, after gestational week 36 twice a week	Not described
**Lv et al (2012) [50]**	150<br>71<br>(47%)	SMS reminders about how to manage asthma	Appointment and medication reminders	Twice a day	At 10:00 a.m. and 8:00 p.m.
**Mao et al (2008) [55]**	100<br>100<br>(100%)	Automated text message reminders about medication	Medication reminder	1 text message	10 min before the medication was due to be taken
**Milne (2010) [68]**	N/a<br>112194[a]<br>N/a	SMS and email last minute reminders	Appointment reminder	1 reminder	2 days in advance of the appointment
**Montes et al (2012) [51]**	254<br>251<br>(99%)	SMS reminders to take medication	Medication reminder	1 message daily	At 11 a.m. or 2 p.m.
**Nundy et al (2013) [54]**	19<br>18<br>(95%)	Automated text messages	Appointment and medication reminders	Daily, semiweekly or weekly	Not described
**Nundy et al (2013) [20]**	27<br>6<br>(22%)	Automated SMS self-care reminders and educational messages	Appointment and medication reminders	Appointment: 48 hours before and the day of the appointment<br>Medication: daily	Not described

**Pena-Robichaux et al (2010) [31]**	27<br>25<br>(93%)	Automated text messages	Treatment reminders and educational messages	1 message daily: treatment reminders 3 times a week and educational information messages 4 times a week	7-9 a.m. or 4-6 p.m.
**Perry (2011) [71]**	N/a<br>447<br>N/a	Automatic SMS appointment reminders	Appointment reminder	The day before the appointment	Not described
**Pijnenborg et al (2007) [30]**	8<br>5<br>(63%)	SMS text messages were used as prompts to remind participants of their daily activities and appointments	Appointment and medication reminders	According to the developed reminding schedule	According to the developed reminding schedule
**Pijnenborg et al (2010) [83]**	62<br>47<br>(76%)	Web-based SMS text messages to improve patients everyday functioning	Functioning reminders	Two SMS text message prompts for each goal	An hour and 10 min before the goal should take place
**Pop-Eleches et al (2011) [46]**	431<br>428<br>(99%)	Automated text message reminders (short and long messages)	Medication reminder	Daily or weekly	At 12 p.m.
**Prasad & Anand (2012) [74]**	N/a<br>206<br>N/a	SMS reminder	Appointment reminder	1 reminder	24 h prior to the appointment and also the day of the appointment
**Rodrigues et al (2012) [40]**	150<br>141<br>(94%)	Mobile phone reminders: an interactive voice response (IVR) call + a non interactive neutral picture delivered as a short message service (SMS)	Medication reminder	Both components once a week on separate days of the week	Time chosen by the participant
**Shaw et al (2013) [22]**	16<br>14<br>(88%)	Text messaging application for sustaining recent weight loss	Preventive and promotive messages (reminders)	Daily	At 9 a.m.
**Sidney et al (2012) [41]**	150<br>139<br>(93%)	Mobile phone reminders: an interactive voice response (IVR) call + a non interactive neutral picture delivered as a short message service (SMS)	Medication reminder	Once a week (SMS 3 days after the call)	At pre-arranged time

**Sims et al (2012) [75]**	N/a<br>1256<br>N/a	Automated SMS reminders	Appointment reminder	7 and 5 or 7 and 3 days before the appointment	At 6 p.m.
**Strandbygaard et al (2010) [43]**	26<br>22<br>(85%)	SMS reminders to take medication	Treatment adherence	1 message daily	At 10 a.m.
**Taylor et al (2012) [76]**	679<br>675<br>(99%)	SMS reminders (automated or manual)	Appointment reminder	2 days (or the day) before the appointment	Not described
**Ting et al (2012) [52]**	79<br>70<br>(89%)	Automated text message reminders	Appointment and medication reminders	7, 3 and 1 day(s) before the appointment and daily medication adherence reminders once or twice a day	At a set time of the day according to medication intake
**Vervloet et al (2012) [81]**	119<br>104<br>(87%)	SMS reminders sent by an electronic medication dispenser	Medication reminder	1 text message	If patients had not opened their medication dispenser within the agreed time period
**Vilella et al (2004) [57]**	N/a<br>4589<br>N/a	Automated SMS reminders	Vaccine reminder	Few days before the date foreseen the next vaccination dose	Not described

Sample[a]=appointment
N/a=Not available
