# Supplementary material for: Use of Mobile Phone Text Message Reminders in Health Care Services: A Narrative Literature Review
Source: J Med Internet Res. 2014 Oct 17;16(10):e222. doi: 10.2196/jmir.3442 (PMC4211035; doi:10.2196/jmir.3442)
Supplement: Supplementary file 3 [file jmir_v16i10e222_app3.pdf]

Author (year)	Outcome measure	Instruments	Outcomes
**Anhøj & Møldrup (2004) [39]**	Patient compliance with an SMS diary	Response rate for requested diary data	↑
**Altuwaijri et al (2012) [72]**	Appointment non-attendance	Reduction rate of non-attendance	↓
**Arora et al. (2012) [32]**	Effectiveness: Medication adherence	MMAS (Morisky Medication Adherence Scale)	↑
**Balato et al (2012) [37]**	Treatment adherence	A multiple-choice question,<br>7 day calendar of adherent days	↑
	Disease severity	PASI (Psoriasis Area Severity Index),<br>BSA (body surface area),<br>PGA (Physician Global Assessment),<br>SAPASI (Self-Administered Psoriasis Area Severity Index)	↓
	Quality of life	DLQI (Dermatology Life Quality Index)	↑
**Boker et al (2012) [47]**	Medication adherence	Self-reported adherence,<br>MEMS (Medication Event Monitoring System)	↓
	Disease severity	Eg digital photographs,<br>Patient Global Assessment scale	↓
**Bos et al (2005) [58]**	Appointment attendance	Attendance rate	⇔
**Bourne et al (2011) [69]**	Re-testing rate	Re-testing rate	↑
**Brannan et al (2011) [56]**	Appointment non-attendance	DNA (Did not attend) -rate	↓
**Branson et al (2011) [70]**	Appointment attendance	Attendance rate	↑
**Chen et al (2008) [61]**	Appointment attendance	Attendance rate	↑
**da Costa et al (2010) [67]**	Appointment non-attendance	Non-attendance rate	↓
**da Costa et al (2012) [80]**	Medication adherence	Self-report,<br>pill counting,<br>MEMS (Medication Event Monitoring System)	↑
**Dick et al (2011) [44]**	Treatment adherence	Self-report: estimation of the number of medication doses missed	↑
**Downer et al (2005) [59]**	Failure to attend (FTA)	FTA (Failure to attend) -rate	↓
**Downing et al (2013) [77]**	Re-testing	Re-testing rate	↑
**Dowshen et al (2012) [48]**	Medication adherence	VAS (Visual analog scale), adherence questionnaire	↑

**Fairhurst & Sheikh (2008) [62]**	Non-attendance	Non-attendance rate	↓
**Fischer et al (2012) [73]**	Appointment attendace	Cancellation rate, no-show rate	⇔
**Foley & O'Neill (2009) [64]**	Appointment non-attendance	Appointment attendance analysis	↓
**Foreman et al (2012) [82]**	Medication adherence	PDC (Proportion of days covered)	↑
**Franklin et al (2006) [35]**	Treatment adherence	Blood test (Hba1c),<br>VAS (Visual analog scale)	↑
	Self-efficacy	SED (self-efficacy for diabetes score),<br>DKN (diabetes knowledge score),<br>DSSI (diabetes social support interview)	↑
**Furberg et al (2012) [21]**	Medication adherence	Medication adherence question via two-way text message	N/a
**Granholm et al (2012) [49]**	Medication adherence	Daily ambulatory monitoring assessment	↑
	Social interactions	Daily ambulatory monitoring assesment	↑
	Severity of hallucinations	Daily ambulatory monitoring assessment	↓
**Guy et al (2013) [78]**	Re-screening	Re-screening rate	↑
**Hanauer et al (2009) [36]**	Blood glucose monitoring	Reminder system usage	↑
**Hardy et al (2011) [45]**	Adherence to antiretroviral therapy	Self report,<br>pill count,<br>MEMS (Medication Event Monitoring System),<br>CAS (composite adherence score)	↑
**Hou et al. (2010) [42]**	Oral contraceptive pill adherence	Electronic monitoring device, diary of pill taking	⇔
**Kollman et al (2007) [79]**	Treatment adherence	Blood test	↑
**Koshy et al (2008) [63]**	Appointment non-attendance	Non-attendance rate	↓
**Leong et al (2006) [60]**	Appointment attendance	Attendance rate	↑
	Costs of text messages	Cost-effectiveness analysis	↓
**Lewis et al (2013) [53]**	Medication adherence	Medication adherence question via two-way text message and survey	↑
**Liew et al (2009) [65]**	Non-attendance	Non-attendance rate	↓
**Ludlow et al (2009) [66]**	Delays in blood monitoring	Number of days of delays	↓
**Lund et al (2012) [34]**	Skilled delivery attendance	Patient register analysis	↑
**Lv et al (2012) [50]**	Perceived control of asthma	PCAQ-6 (six item perceived control of asthma questionnaire)	↑

	Quality of life	AQLQ(S) (Asthma-Specific Quality of Life)	↑
	Follow-up adherence	Follow-up adherence rate	↑
	Medication compliance	Medication compliance rate	↑
	Emergency department visits	Emergency department visit data	↓
**Milne (2010) [68]**	Appointment non-attendance	DNA (Did not attend) -rate	↓
**Montes et al (2012) [51]**	Medication adherence	MAQ (Morisky Green Adherence Questionnaire)	↑
	Severity of illness	CGI-SCH (Clinical Global Impression - Schizophrenia Scale)	↓
	Attitude towards medication	DAI-10 (Drug Attitude Inventory)	↑
	Awareness of illness	SUMD (Scale to Assess Unawareness of Mental Disorder)	⇔
	Quality of life	EQ-5D (EuroQol)	↑
**Nundy et al (2013) [54]**	Organizing diabetes care	Interviews	↑
	Self management	Interviews	↑
**Nundy et al (2013) [20]**	Maintenance and self-management	SCHFI (The Self-Care of Heart Failure Index)	↑
**Pena-Robichaux et al (2010) [31]**	Treatment adherence	7-day recall calendar,<br>a multiple-choice question in a survey about the adherence	↑
	Self care behavior	Structured questionnaire	↑
	Skin severity	SCORAD (SCOring Atopic Dermatitis index)	↑
	Quality of life	DQLI (Quality of Life Index)	↑
**Perry (2011) [71]**	Failed attendance	Failed attendance rate	↓
**Pijnenborg et al (2007) [30]**	Medication compliance	Daily target achievement recording (percentage of goals achieved)	↑/↓
	Attendance	Daily target achievement recording (percentage of goals achieved)	↑/↓
**Pijnenborg et al (2010) [83]**	Achievement of goals	Percentage of goals achieved	↑/↓
**Pop-Eleches et al (2011) [46]**	Adherence to antiretroviral treatment	MEMS (Medication event monitoring system)	↑
**Prasad & Anand (2012) [74]**	Appointment attendace	Attendance rate	↑
**Rodrigues et al (2012) [40]**	Adherence to antiretroviral therapy	Pill count	↑
**Shaw et al (2013) [22]**	Adherence to nutrition and exercise	Self-report (weight)	↑
**Sims et al (2012) [75]**	Appointment attendace	Attendance rate	↑
**Strandbygaard et al (2010) [43]**	Treatment adherence	Adherence rate	↑
**Taylor et al (2012) [76]**	Appointment non-attendance	Non-attendance rate	↓
**Ting et al (2012) [52]**	Appointment adherence	Attendance rate	↑

	Medication adherence	MASRI (Medication Adherence Self-Report Inventory), blood test, pharmacy refill information	⇔
**Vervloet et al (2012) [81]**	Adherence to oral antidiabetics	RTMM (Real Time Medication Monitoring)	↑
**Vilella et al (2004) [57]**	Compliance with vaccination	Compliance rate	↑

↑ Outcome increased or improved
↓ Outcome decreased
⇔ No impact on the outcome
N/a=Not available
